# Recent Progress in Silicon-Based On-Chip Integrated Infrared Photodetectors

**DOI:** 10.3390/s26041125

**Published:** 2026-02-09

**Authors:** Yu He, Hongling Peng, Peng Cao, Zeyu Wang, Jiaqi Wei, Chunxu Song, Wanhua Zheng, Qiandong Zhuang

**Affiliations:** 1 Laboratory of Solid-State Optoelectronics Information Technology, Institute of Semiconductors, Chinese Academy of Sciences, Beijing 100083, China; heyu@semi.ac.cn (Y.H.); hlpeng@semi.ac.cn (H.P.); pengkt11@semi.ac.cn (P.C.); zywang@semi.ac.cn (Z.W.); jqwei@semi.ac.cn (J.W.); songchunxu@semi.ac.cn (C.S.); whzheng@semi.ac.cn (W.Z.); 2 College of Materials Science and Opto-Electronic Technology, University of Chinese Academy of Sciences, Beijing 100049, China; 3 College of Electronic and Communication Engineering, University of Chinese Academy of Sciences, Beijing 100049, China; 4 Physics Department, Lancaster University, Lancaster LA1 4YB, UK

**Keywords:** on-chip integration, silicon photonics, infrared photodetectors, heterogeneous integration

## Abstract

Infrared (IR) photodetectors are indispensable to modern optoelectronic systems, ranging from night vision imaging, surveillance, and industrial process control to environmental monitoring and medical diagnostics. However, traditional detectors based on bulk semiconductors are constrained by prohibitive fabrication costs and the stringent requirement for bulky cryogenic cooling, which severely hinders their widespread deployment in Size, Weight, and Power (SWaP)-sensitive scenarios. Silicon-based on-chip integration, leveraging compatibility with mature CMOS processes, has emerged as a transformative paradigm. It enables the realization of fully functional photonic integrated circuits (PICs) capable of on-chip sensing and high-speed data transmission, offering a pathway toward miniaturized and cost-effective architectures. This article provides a review of recent progress in silicon-based infrared photodetectors across three core material systems: Group IV (Ge/GeSn), III–V compounds, and two-dimensional (2D) materials. In the end, we offer an outlook on the development trends of next-generation intelligent sensing systems driven by optoelectronic convergence.

## 1. Introduction

The infrared (IR) spectrum, which includes short-wave (SWIR, 1–3 μm), mid-wave (MWIR, 3–5 μm) and long-wave (LWIR, 8–14 μm) infrared regions, occupies a position of paramount importance in modern optoelectronics [1,2,3]. This significance is primarily attributed to the unique molecular absorption spectra and the peak thermal emission of room-temperature objects within this band [4]. Consequently, infrared technology has become indispensable in a diverse range of applications, including night vision [5,6], thermal imaging [7,8], environmental monitoring [9,10], industrial process control [11], and medical diagnostics [12,13]. The infrared photodetector serves as the core component of these systems, which function as a fundamental device for photoelectric conversion. Their performance characteristics essentially govern the sensitivity and bandwidth of the entire sensing architecture.

For several decades, high-performance infrared detection has been dominated by narrow-bandgap bulk semiconductors, particularly Mercury Cadmium Telluride (HgCdTe) [14,15] and Indium Antimonide (InSb) [16]. Owing to their near-unity quantum efficiency, these materials have been solidified as the benchmark for high-performance scientific instrumentation [17]. Nevertheless, despite their exceptional optoelectronic characteristics, these incumbent materials are subject to fundamental constraints that impede their widespread implementation in cost-sensitive and portable applications. Specifically, the epitaxial growth and fabrication of HgCdTe are notably complex and resource-intensive, primarily due to the inherent toxicity of its constituent elements and significant lattice mismatch with available substrates [18,19]. Furthermore, these detectors typically necessitate sophisticated cryogenic cooling systems to mitigate thermally generated dark currents [20], a prerequisite for achieving background-limited infrared photodetector (BLIP) performance. Consequently, these factors severely restrict the integration of such detectors into emerging platforms where SWaP constraints are paramount, particularly portable devices and distributed sensing networks.

To address these fundamental constraints, significant research efforts have increasingly focused on on-chip integration as a promising strategy for next-generation infrared sensing. The integration of infrared sensing materials onto silicon platforms enables the exploitation of well-established and highly scalable Complementary Metal-Oxide-Semiconductor (CMOS) fabrication processes. This shift toward on-chip solutions yields several distinct advantages, including significant miniaturization, enhanced modularity in system design, and the cost-effective realization of high-density, large-format focal plane arrays (FPAs) [21].

Moreover, the rapid advancement of silicon photonic (SiPh) establishes a robust platform for the development of high-performance infrared sensing architectures [22,23,24]. Silicon photonic integration facilitates efficient coupling between guided optical modes and active absorption layers, thereby enhancing light-matter interactions and enabling the development of sophisticated, multi-functional photonic integrated circuits (PICs) [25,26]. The integration of diverse material systems, such as Germanium (Ge), III–V compounds, and emerging low-dimensional materials, into a silicon-compatible framework capitalizes on the inherent economies of scale of CMOS technology. This multifaceted integration enables on-chip detectors to transition from specialized high-end instrumentation toward pervasive, cost-effective sensing ecosystems.

In this article, we provide a comprehensive review of the state-of-the-art advancements in silicon-based integrated infrared photodetectors, emphasizing their synergistic integration with the silicon photonics ecosystem. The discussion is systematically organized based on material systems, specifically evaluating the progress and challenges associated with Ge-based, III–V compound semiconductor, and two-dimensional (2D) material-based photodetectors.

## 2. Ge-on-Si Photodetectors

To overcome the inherent limitations of conventional Si-based devices, the Group IV element Ge has emerged as a leading candidate [27]. Ge exhibits several distinct advantages. Most notably, compared to the 1.1 eV indirect bandgap of Si, Ge features a narrower bandgap of approximately 0.8 eV for specific strains, which facilitates a high optical absorption coefficient across the telecommunication C and L bands. Furthermore, Ge possesses enhanced carrier transport properties, with electron and hole mobilities that substantially outperform those of Si [28]. Beyond its physical merits, Ge fabrication is intrinsically compatible with silicon-based platforms [29], facilitating monolithic integration within standard CMOS foundries. In recent years, research on Ge-on-Si photodetectors (PDs) has focused on achieving performance breakthroughs, specifically addressing the trade-off between bandwidth and responsivity, as well as enabling high-power handling and extending the detection range toward longer wavelengths.

### 2.1. High Speed Ge Photodetectors

To achieve high-speed Ge-on-Si photodetectors, it is essential to optimize the two primary bandwidth-limiting factors: carrier transit time and RC delay. A key challenge in Ge-on-Si PD design lies in the intrinsic trade-off between these two factors: while a thinner intrinsic Ge layer reduces transit time, it simultaneously increases junction capacitance, thereby exacerbating the RC delay. To further extend the frequency response, gain peaking techniques are utilized to mitigate RC parasitics [30,31]. Additionally, optimized doping profiles refine the internal electric field, ensuring carriers reach saturation velocity for ultra-fast operation [32,33]. Based on their device architectures, Ge-on-Si PDs are generally classified into two categories: vertical PIN (V-PIN) and lateral PIN (L-PIN) structures.

#### 2.1.1. Vertical PIN (V-PIN)

V-PIN represents the earliest and most established architecture and is typically fabricated using a mesa process. In a V-PIN configuration, the p-type region, the intrinsic Ge absorption layer, and the n-type region are vertically stacked perpendicular to the substrate surface. The thickness of the intrinsic layer governs the fundamental interplay between responsivity and bandwidth [34]. An increase in layer thickness augments optical absorption and responsivity; however, the prolonged carrier transit time inherently restricts the bandwidth.

**Figure 1:**
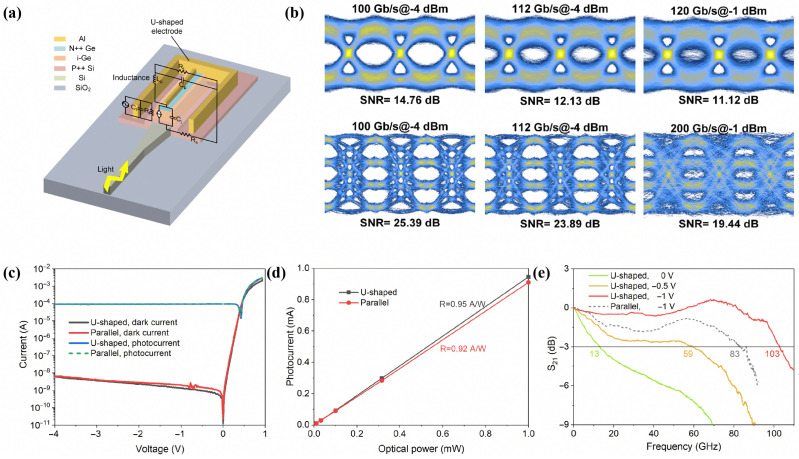
(**a**) Structure of the U-shaped electrode PDs. (**b**) Measured eye diagrams. (**c**) I–V characteristics of the two kinds of PDs. (**d**) Photocurrent with the input optical power at −1 V. (**e**) Normalized $S_{21}$ parameters of the PDs. Reproduced with permission from Ref. [32].

To circumvent the inherent trade-off between responsivity and bandwidth, Shi et al. reported a vertical Ge-on-Si photodetector incorporating a novel U-shaped electrode configuration [32]. This design yielded a 36% reduction in parasitic series resistance without a commensurate increase in junction capacitance, thereby alleviating the RC-related constraints typical of conventional devices. As illustrated in Figure 1, the device attained an ultra-high 3-dB bandwidth of 103 GHz at a −1 V bias, while concurrently maintaining a high responsivity of 0.95 A/W and a negligible dark current of 1.3 nA.

**Figure 2:**
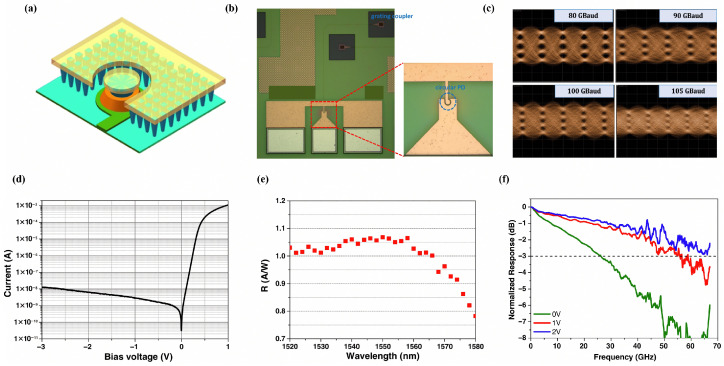
(**a**) Structure of the PD with light-trapping architecture. (**b**) Optical micrograph of the fabricated circular Ge PD. (**c**) BTB eye diagram of PAM-4 signals. (**d**) Dark current versus voltage. (**e**) Normalized responsivity at different wavelengths. Reproduced with permission from Ref. [34].

An alternative strategy to circumvent this trade-off involves the incorporation of light-trapping structures. In 2022, Chen et al. proposed and demonstrated a vertical Ge photodetector leveraging a circular light-trapping architecture [34]. This design leverages tangential coupling to confine the optical beam, inducing it to circulate along the periphery of a circular Ge mesa with a predefined radius. This mechanism substantially elongates the effective absorption path, particularly for long-wavelength photons. As shown in Figure 2, experimental results reveal that the detector attained a 3 dB bandwidth of 67 GHz at −2 V, while exhibiting a high responsivity of 1.05 A/W across the 1520–1560 nm range. Furthermore, the device successfully facilitated PAM-4 signal transmission at a data rate of 240 Gb/s.

#### 2.1.2. Lateral PIN (L-PIN)

In contrast to vertical architectures, L-PIN structures are characterized by p- and n-doped regions disposed on opposite sides of the intrinsic Ge layer, facilitating carrier transport parallel to the substrate. This configuration enables the independent optimization of electrode spacing and optical absorption length [35]. Moreover, the minimized junction area inherent in lateral designs substantially mitigates parasitic capacitance, thereby alleviating RC-related constraints and facilitating high-bandwidth performance.

**Figure 3:**
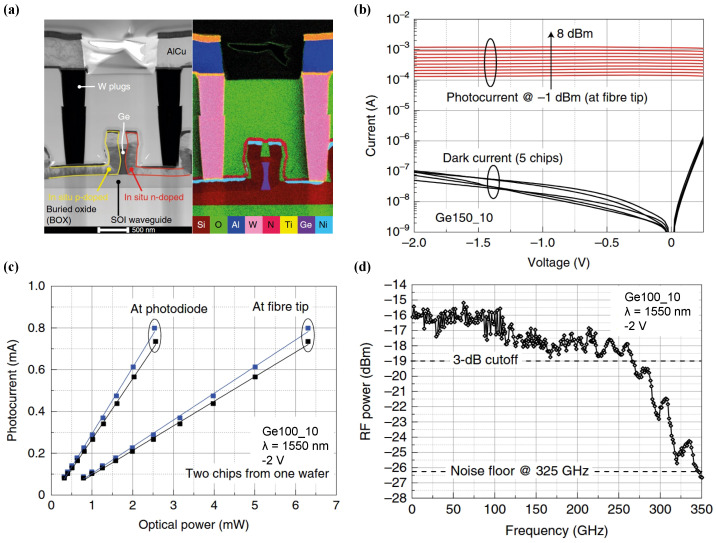
(**a**) Cross-sectional image obtained by STEM and EDX. (**b**) Dark currents under an optical input power sweep. (**c**) Photocurrent plotted versus optical power for internal/external responsivities with linear fits. (**d**) Frequency responses from a heterodyne measurement. Reproduced with permission from Ref. [35].

Building upon these architectural advantages, Lischke et al. set a new benchmark for Ge-on-Si photodetector bandwidth by pioneering an innovative Germanium Fin structure [35]. Drawing inspiration from Fin Field-Effect Transistor (FinFET) design principles, this work leveraged fabrication processes with sub-100 nm feature sizes coupled with in situ-doped epitaxial growth. The width of the germanium fin is scaled down to a mere 60 nm at its narrowest point, a dimension that substantially curtails the carrier transit distance and time. Consequently, this aggressive scaling facilitated an unprecedented optoelectronic response, with the device demonstrating a 3 dB bandwidth surpassing 265 GHz, as illustrated in Figure 3d. These high-speed attributes were coupled with an internal responsivity of 0.3 A/W at 1550 nm, while the dark current remained consistently within the range of 100–200 nA. This milestone underscores the industrial viability of mass-producing ultra-fast photodetectors for sub-400 GBaud applications within standard 8-inch CMOS foundries.

Beyond academic research, the industry has realized high-performance detectors through alternative development routes that prioritize process scalability. Utilizing the foundry-based GlobalFoundries 300 mm monolithic CMOS silicon photonics platform, Bian et al. reported a Ge PIN photodetector with exceptional speed [36]. The architecture incorporates optimized doping profiles and chamfered Ge layer designs to mitigate electric field crowding. Specifically, the detector attains an optoelectronic bandwidth surpassing 135 GHz alongside a high responsivity of 0.96 A/W. This achievement exemplifies a successful synergy between state-of-the-art performance and industrial-scale manufacturing feasibility.

**Figure 4:**
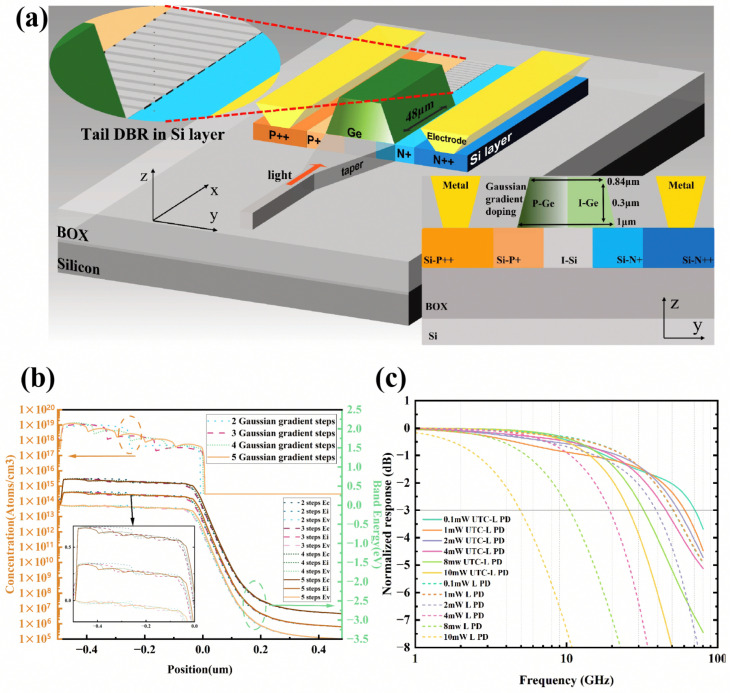
(**a**) Structure of the waveguide-coupled uni-traveling-carrier lateral photodetector. (**b**) Doping distribution and band structure near the Ge/Si junction at different Gaussian gradient steps. (**c**) Small-signal optoelectronic frequency response. Reproduced with permission from Ref. [33].

To support high-order modulation formats, photodetectors are required to sustain high-incident optical powers while maintaining a linear response without reaching saturation. Conventional PIN structures are susceptible to space-charge effects under high-power illumination, which induces electric field screening and leads to a dramatic degradation in both bandwidth and linearity. UTC photodetectors provide an effective approach to mitigating space-charge effects, enabling superior high-power performance by decoupling electron and hole transport. In 2025, Wang et al. reported a lateral UTC Ge-on-Si photodetector incorporating a Gaussian-profiled gradient doping and a rear distributed Bragg reflector (DBR) architecture [33]. The architecture utilizes a step-wise Gaussian gradient p-type doping profile within the Ge absorption layer as shown in Figure 4a to induce a robust built-in electric field. Figure 4c shows that the structure achieves up to 70 GHz bandwidth and maintains a 25 GHz high-speed response under 10 mW optical input power. The saturation power reaches approximately 30 mW. This field accelerates electron transport via drift and effectively suppresses hole accumulation at high optical powers, thereby mitigating bandwidth degradation associated with space-charge screening effects.

**Figure 5:**
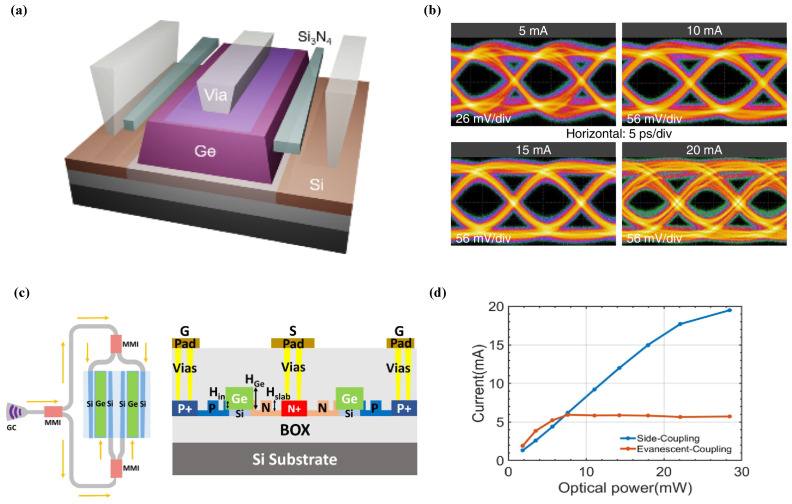
(**a**) Structure of the Ge-on-Si PD with double lateral ${\mathrm{Si}}_{3}N_{4}$ waveguides. (**b**) 60 Gbit/s NRZ eye diagrams. Reproduced with permission from Ref. [37]. (**c**) Structure of the side-coupling architecture. (**d**) Responsivity–optical power curves for side-coupling (blue) and evanescent-coupling (orange) architectures. Reproduced with permission from Ref. [38].

An alternative approach to overcoming this challenge involves expanding the optical mode area or utilizing multi-waveguide coupling to reduce the optical power density. For instance, Li et al. demonstrated a novel Ge-on-Si photodetector incorporating double lateral silicon nitride (${\mathrm{Si}}_{3}N_{4}$) waveguide coupling [37]. This architecture employs dual lateral coupling to facilitate a homogeneous distribution of the optical field within the Ge absorption region as schematically illustrated in Figure 5a. Consequently, localized carrier accumulation is mitigated, effectively enhancing transport efficiency and linearity during high-power operation. Similarly, Cao et al. reported a side-coupling architecture that distributes the input light into four branches via cascaded multimode interference (MMI) couplers [38]. This configuration leverages adiabatic side-coupling to facilitate the gradual evanescent transfer of light into two parallel Ge absorption waveguides. Notably, even under a high input power of 28 mW, the device maintains a robust responsivity of 0.68 A/W and attains a saturation photocurrent in excess of 19.5 mA as shown in Figure 5d. Furthermore, the detector exhibits a 3 dB bandwidth surpassing 40 GHz under small-signal conditions. Its operational reliability at high power is further evidenced by the successful reception of 25 Gbps signals at a 16 mA photocurrent and 50 Gbps signals at 8.5 mA.

From a systems engineering perspective, the pursuit of high bandwidth is essentially a trade-off between physical limits and engineering margins. Achieving high bandwidth often requires thinning the absorption layer, which directly reduces quantum efficiency and imposes a ceiling on sensitivity. Simultaneously, a wider spectral window inevitably introduces higher thermal and shot noise. This elevates the noise floor and further compresses the dynamic range. At the hardware level, high-frequency signals are extremely sensitive to parasitic parameters, forcing packaging to evolve from simple electrical interconnects into complex microwave engineering to ensure impedance matching. To maintain signal integrity at high speeds, back-end amplifiers must operate at high bias currents. The resulting power consumption and thermal accumulation not only challenge energy efficiency but may also degrade dark current performance due to temperature-induced drift.

### 2.2. Ge-on-Si Avalanche Photodetectors

Compared to conventional PIN photodetectors, avalanche photodiodes (APDs) exhibit superior sensitivity owing to their inherent internal gain mechanism [39]. The incorporation of a dedicated multiplication region facilitates impact ionization, which substantially enhances the responsivity of the receiver and enables the detection of ultra-weak optical signals. Ge-on-Si APDs leverage the high absorption coefficient of Ge in the near-infrared regime alongside the low-noise multiplication properties of Si [39,40]. This heterostructure represents an optimal material synergy, as Si possesses an exceptionally low *k*—the ratio of hole-to-electron impact ionization coefficients [41]. A low *k* value indicates that electrons more easily receive impact ionization than holes. Consequently, stochastic gain fluctuations are minimized, suppressing the primary source of excess noise. Despite the 4.2% lattice mismatch between Ge and Si, progress in epitaxial growth have substantially suppressed the dark current in Si-Ge APDs [39]. The gain-bandwidth product (GBP) serves as a key figure of merit for evaluating APD performance, encapsulating the fundamental trade-off between multiplication-induced sensitivity enhancement and the intrinsic constraints on response speed.

**Figure 6:**
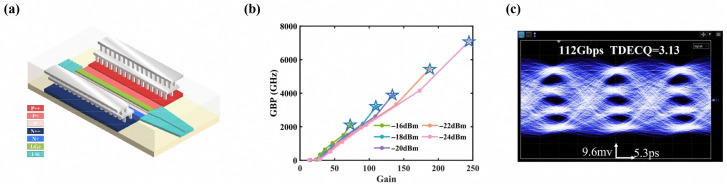
(**a**) Structure of the SACM APD. (**b**) The GBP of the APD. (**c**) Eye diagrams measured at data rates of 112 Gbps PAM4. Reproduced with permission from Ref. [43].

In recent years, the GBP of Si-Ge APDs has achieved significant breakthroughs. At ECOC 2024, Cao et al. proposed a lateral Ge/Si APD featuring non-uniform electric field profiling [42]. By strategically incorporating lightly doped *p*-type and *n*-type regions, this design establishes a graded electric field distribution within the multiplication layer to optimize avalanche dynamics. This mechanism exploits the dead-space effect to effectively suppress the stochastic nature of impact ionization. Such spatial non-locality leads to a significant reduction in the effective ionization coefficient ratio ($k_{eff}$), reaching levels below the intrinsic limit of bulk Silicon. This work highlights a remarkably high GBP of 3036 GHz at a low optical input of −24 dBm. Moreover, the prototype demonstrated exceptional signal integrity for 50 Gbps NRZ detection. At OFC 2025, Cheng et al. demonstrated a lateral Separate Absorption, Charge, and Multiplication (SACM) Si-Ge APD [43], and the device structure is detailed in Figure 6a. This design incorporates ten pairs of DBRs to facilitate secondary absorption, which enhances the responsivity by 35%. Furthermore, through precise electric field regulation, the device achieved a record-breaking GBP of 7078 GHz at an input power of −24 dBm, as shown in Figure 6. System-level characterization confirmed that the device successfully facilitated 112 Gbps PAM4 signal transmission, underscoring its viability for high-capacity optical interconnects.

**Figure 7:**
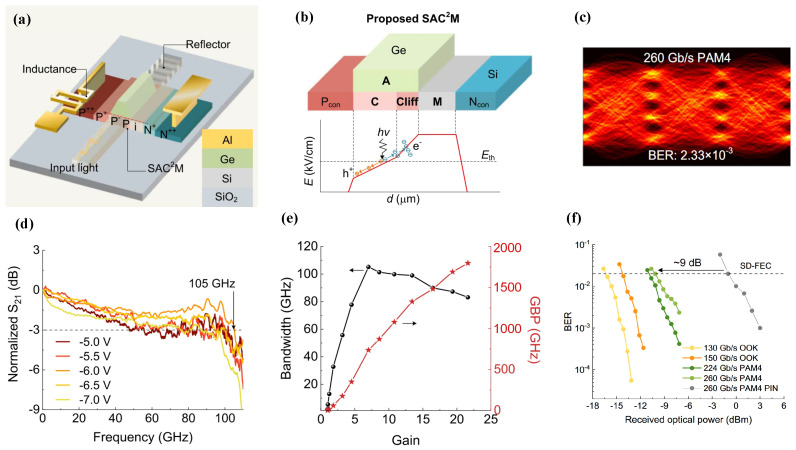
(**a**) Structure of the $SAC^{2}M$ APD. (**b**) The cross-section of junction region and the theoretical electric field distribution. (**c**) Eye diagrams measured at data rates of 260 Gbps PAM4. (**d**) Frequency responses under −22 dBm. (**e**) The measured bandwidth and the GBP of the APD. (**f**) Bit error rate results for the on-off keying and four-level pulse amplitude modulation signals under different received optical power. Reproduced with permission from Ref. [44].

While APDs have traditionally been constrained by limited response speeds, Shi et al. shattered this bottleneck by demonstrating a single-carrier multiplication APD architecture enabled by precision electric field gradient engineering [44]. This research pioneered a Separated Absorption-Charge-Cliff-Multiplication ($SAC^{2}M$) architecture, and Figure 7b shows the device structure. By profiling a longitudinal electric field gradient within the ultra-thin Ge absorption layer, this architecture dictates an electron-dominated asymmetric multiplication. This configuration drastically suppresses the avalanche buildup time, enabling ultra-high-speed operation. Empowered by on-chip inductive peaking technology, the device delivers a state-of-the-art bandwidth of 105 GHz alongside an unprecedented GBP of 4800 GHz, setting a new benchmark for Si-Ge APDs. As illustrated in Figure 7f, system-level characterization during 260 Gbps PAM4 transmission experiments demonstrated a 9 dB sensitivity enhancement compared to conventional unity-gain detectors.

High-gain operation in Ge-on-Si APDs is restricted by substantial excess noise, a major obstacle for long-haul and weak-signal sensing. This excess noise factor (*F*) reduces the signal-to-noise ratio (SNR), consequently limiting both the bit error rate and effective detection range. The SACM structure serves as the core strategy for noise suppression in Ge-on-Si APDs through meticulous electric field management [45,46]. The design ensures the Ge absorption layer sustains a field strong enough for high-speed carrier drift but low enough to avoid impact ionization. Simultaneously, a high-intensity field is concentrated in the Si layer to trigger efficient avalanche multiplication. Furthermore, the dead-space effect in ultra-thin multiplication layers is leveraged to suppress noise [44]. Fleming et al. demonstrated a surface-normal illuminated pseudo-planar Ge-on-Si APD that effectively suppresses edge breakdown through optimized electric field distribution [47]. By utilizing a 2 µm thick Ge absorption layer to enhance responsivity at 1550 nm and a 1.4 µm thick Si multiplication layer to reduce the $k_{eff}$, the device achieved a maximum avalanche gain of 101 at 1550 nm. Furthermore, the device exhibited a record-low excess noise factor (*F*) of 3.1 at a gain of 20.

### 2.3. GeSn Photodetectors

While Ge exhibits excellent performance within the telecommunication C-band, its direct bandgap absorption edge is situated near 1.55 μm. Beyond this threshold, the absorption coefficient experiences a precipitous decline, limiting its efficacy for longer wavelength applications. To circumvent this constraint, research has shifted toward the non-equilibrium incorporation of tin (Sn) into the Ge lattice. The resulting GeSn alloys enable precise bandgap engineering across an extensive spectral range, spanning the SWIR, MWIR, and, potentially, the LWIR regimes [48]. Table 1 summarizes the reported GeSn photodetectors.

More importantly, the GeSn band structure transforms into a direct bandgap configuration when the Sn content exceeds a specific threshold, enabling efficient, phonon-less optical transitions for long-wavelength detection [49]. Furthermore, the material exhibits exceptional compatibility for monolithic integration with standard Si-CMOS fabrication workflows. Consequently, GeSn is hailed as a premier candidate for achieving cost-effective and large-scale silicon-based infrared PICs.

For GeSn photodetectors, dark current is the primary factor determining detectivity ($D^{*}$) and noise equivalent power. The dark current in GeSn photodetectors is primarily dominated by Shockley–Read–Hall recombination induced by high threading dislocation densities from lattice mismatch, and diffusion current exacerbated by the narrowed bandgap as Sn content increases. Under high reverse bias or high doping concentrations, trap-assisted tunneling (TAT) and band-to-band tunneling (BTBT) become dominant leakage paths due to the material’s low effective mass and small energy gap. Moreover, the impact of Sn segregation on the device’s electrical performance cannot be overlooked. Sn atoms tend to migrate toward the growth front, leading to surface segregation or Sn-rich clusters. These regions act as metallic shunting paths and recombination centers within the depletion region, significantly increasing leakage current. Furthermore, Sn segregation at hetero-interfaces introduces deep-level traps that facilitate defect-assisted transport, such as TAT.

The advancement of GeSn infrared photodetector performance is fundamentally contingent upon the synthesis of high-quality epitaxial layers with high Sn-incorporation levels. The primary challenge in GeSn epitaxy lies in achieving high Sn concentrations without precipitation, a task complicated by Sn’s limited solubility [50] and large atomic footprint relative to the Ge host lattice. Molecular beam epitaxy (MBE) [51,52] and chemical vapor deposition (CVD) [53,54] represent the two predominant epitaxial methodologies for the synthesis of GeSn alloys. These platforms are particularly valued for their ability to maintain low-temperature growth regimes, which are essential for achieving high Sn-incorporation levels. While MBE offers unrivaled advantages in the growth of ultra-thin layers with atomic-scale precision, CVD is inherently better suited for high-throughput and large-scale industrial manufacturing.

**Figure 8:**
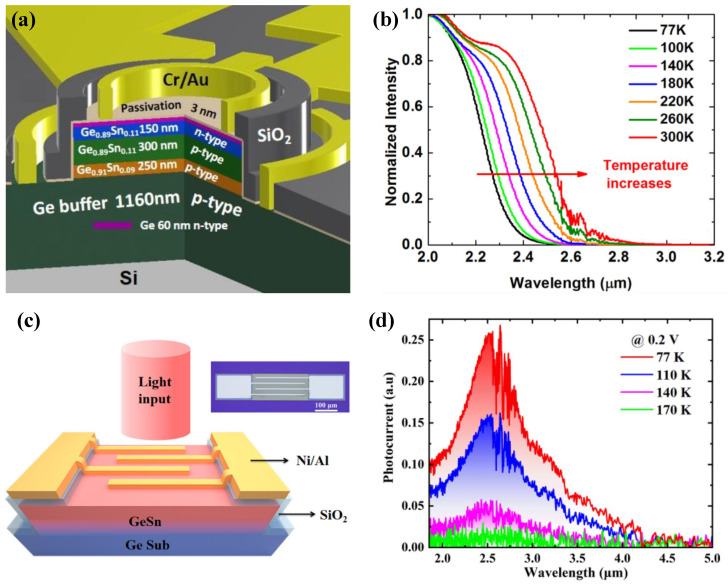
(**a**) Structure of the GeSn PD realized by MBE. (**b**) Photocurrent spectra of the GeSn photodetector. Reproduced with permission from Ref. [55]. (**c**) Device structure schematic diagram. (**d**) Temperature-dependent spectral response. Reproduced with permission from Ref. [56].

Liu et al. demonstrated a Sn-compositionally graded buffer architecture synthesized via MBE [55]. By meticulously modulating the strain evolution during epitaxy, the researchers successfully realized high-crystalline-quality, fully relaxed GeSn thick films with a Sn fraction as high as 16.3%. This methodology effectively mitigated strain-induced Sn segregation typically associated with lattice mismatch. At an operating temperature of 77 K, Figure 8b shows the resulting detector exhibiting exceptional MWIR performance, with a cutoff wavelength extending to 4.2 μm and a peak responsivity of 0.35 A/W. H. Tran et al. demonstrated the fabrication of GeSn photodetectors on Si substrates leveraging an industry-standard reduced pressure chemical vapor deposition (RPCVD) system [56]. The study showcased a significant range of Sn incorporation levels, spanning from 10.5% to an impressive 22.3%. This work synergistically utilized a spontaneous relaxation enhanced (SRE) growth strategy and innovative germanium oxynitride (GeON) surface passivation to ameliorate the device interfaces and bulk crystal quality. These methodologies successfully extended the cutoff wavelength to 3.65 μm, as shown in Figure 8d. The photodetectors based on GeSn with different Sn content, responsivity, and cut-off wavelength are shown in Table 1.

**Table 1:** Performance summary of GeSn photodetectors.

Sn Content (%)	$J_{\mathrm{dark}}$ (A/cm^2^)	Responsivity (A/W)	Cut-Off Wavelength (nm)	Ref.
3.4	0.025 @-1 V	0.18 @1550 nm	1750	[57]
3.6	0.006 @-1 V	0.71 @1790 nm	2200	[58]
5	0.073 @-1 V	0.18 @1550 nm	1877	[59]
6	0.078 @-1 V	0.45 @1550 nm	2300	[60]
10	22 @-1 V	0.15 @1550 nm	2600	[61]
8	2.7 @-1 V	0.093 @2000 nm	2300	[62]
8	0.016 @-1 V	0.307 @1550 nm	2200	[63]
3	0.3 @-1 V	0.32 @1550 nm	1800	[64]
11	0.0079 @0.1 V	0.32 @2000 nm	2600	[54]
8–13	2.8 @-1 V	0.24 @1550 nm	2800	[65]
11–14.3	0.3 @-1 V	0.11 @2000 nm	3300	[52]

## 3. III–V Compound Semiconductors

Owing to the scalable fabrication capabilities of mature CMOS processes, Group IV materials have emerged as a leading platform for large-scale silicon-based photonic integration. However, the inherent indirect bandgap of Group IV materials continues to stifle their performance in high-efficiency light emission and ultra-fast optoelectronic conversion. Conversely, III–V compound semiconductors are exemplified by materials such as gallium arsenide (GaAs), indium phosphide (InP), and gallium antimonide (GaSb). These compounds afford significantly greater design versatility, primarily through advanced heterostructure engineering and tunable bandgap properties [66,67,68]. Leveraging high mobility and a tunable direct bandgap, III–V materials are ideally suited to address the fundamental performance limits of traditional Group IV photonic devices. Specifically, InGaAs is the benchmark for telecommunication O and C bands due to its high quantum efficiency, while antimonide-based (GaSb/InAsSb) materials enable high-performance detection extending into the MWIR and LWIR. For the heterogeneous integration of III–V devices onto silicon platforms, the predominant methodologies encompass wafer bonding [69,70,71,72,73], hetero-epitaxial growth [74,75,76], and micro-transfer printing (μTP) [77,78,79,80,81].

### 3.1. Bonding

Heterogeneous integration via bonding entails the transfer of III–V wafers or dies onto Si substrates through molecular or chemical interactions. Following the bonding process, the native growth substrate is removed via selective etching or grinding, leaving only the active epitaxial membranes for device fabrication [70]. Based on the interface chemistry, these methods are primarily classified as direct bonding or intermediate-layer bonding.

#### 3.1.1. Direct Bonding

Direct bonding is a widely adopted integration technique that enables the seamless joining of dissimilar materials via intermolecular forces, without the need for intermediate adhesive layers. This methodology serves as the predominant technological vehicle for the mass production of high-performance SiPh platforms by industry leaders, most notably Intel. Direct bonding ensures high optical and thermal performance by providing a seamless interface [71]. To mitigate thermo-mechanical stress arising from the coefficient of thermal expansion (CTE) mismatch between III–V materials and Si, recent efforts have focused on low-temperature annealing. By optimizing plasma activation parameters and utilizing ultra-thin buffer layers, high-strength bonding has been achieved at temperatures between 200 °C and 300 °C [69]. This advancement successfully minimizes residual stress and suppresses wafer warpage during the cooling phase.

Since the 1990s, heterogeneous integration via direct bonding has been extensively explored to combine the high near-infrared absorption of III–V semiconductors with the low excess noise of silicon avalanche regions [82]. Following the successful demonstration of InGaAs-Si APDs with high gain and low noise characteristics [83,84], the research has expanded to integrating diverse III–V absorbers with silicon. This strategy synergizes the high quantum efficiency of III–V materials and silicon’s low-noise multiplication for high-sensitivity detection. For instance, Nallamothu et al. realized the direct bonding of GaAsSb absorbers to silicon substrates, combining high quantum efficiency at 1550 nm with the superior multiplication performance of silicon [85].

Distinct from the aforementioned APD architectures that utilize silicon as the avalanche multiplication layer, another significant research trajectory employs bonding techniques to integrate complete III–V photodetector structures with CMOS-compatible silicon waveguides or readout circuits. Early demonstrations, such as the work by Chen et al. [86], successfully achieved the hybridization of InP-based photodiode arrays with silicon readout circuitry through direct bonding and vertical interconnections. More recently, to meet the demands of coherent transmission networks, Okimoto advanced this hybrid platform by directly bonding III–V photodetectors to silicon waveguides [87]. By introducing a novel air-cladding structure, this configuration optimizes the trade-off between responsivity and bandwidth, thereby facilitating the dense integration of diverse active and passive functionalities on a single receiver chip.

#### 3.1.2. Intermediate-Layer Bonding

While plasma activation effectively reduces the annealing temperature, direct bonding remains predicated on sub-nanometer surface flatness and pristine interfacial cleanliness. Given these stringent constraints, research attention has increasingly pivoted toward intermediate-layer bonding as a process-tolerant alternative. By utilizing a functional interlayer—such as metal, adhesive, or dielectric—this approach significantly relaxes the requirements on surface topography [68].

Among the available interlayer materials, divinylsiloxane-bis-benzocyclobutene (DVS-BCB) stands as the most established adhesive bonding agent. This technique typically involves spin-coating or spray-coating a thin DVS-BCB polymer layer to affix III–V wafers or dies onto pre-patterned Silicon-on-Insulator (SOI) platforms, ensuring both mechanical robustness and optical coupling. Early work by Roelkens et al. validated this approach for the heterogeneous integration of InP-based active components with passive SOI circuits [15]. Building on this foundation, Gassenq et al. subsequently extended the technology to the short-wave infrared regime, successfully demonstrating GaInAsSb photodiodes integrated via BCB bonding with efficient evanescent coupling [88]. In recent years, research has prioritized the precise modulation of DVS-BCB thickness to minimize optical loss. S. Keyvaninia et al. optimized several critical parameters, including spin-coating speeds, solution dilution ratios, and pressure control prior to the curing process [89]. These refinements successfully yielded nanometer-scale uniform adhesive layers. Such a reduction in interlayer thickness facilitated a substantial enhancement in coupling efficiency between the III–V functional layers and the underlying waveguides.

**Figure 9:**
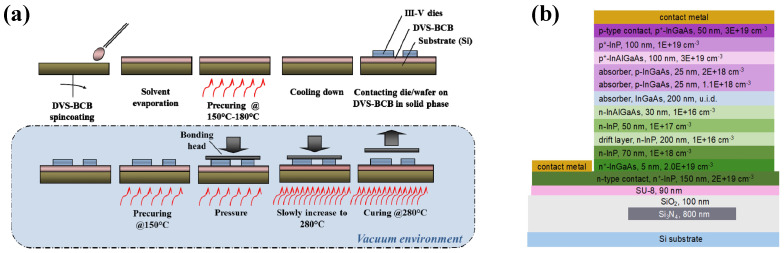
(**a**) Developed bonding process used DVS-BCB. Reproduced with permission from Ref. [89]. (**b**) Cross-sectional view of heterogeneously integrated PD used SU-8. Reproduced with permission from Ref. [92].

Alongside BCB, the epoxy SU-8 represents another pivotal adhesive in heterogeneous integration, favored for its low optical transmission loss and low curing temperature. Leveraging these properties, Shao et al. employed an SU-8 interlayer to bond InGaAs APDs onto SOI substrates, integrating a two-dimensional photonic crystal structure to enhance light absorption [90]. This photon-trapping design enabled the use of a thin 250 nm absorber, achieving a record-high responsivity of 0.75 A/W, equivalent to an external quantum efficiency of 57.2% at 1626 nm while maintaining a low dark current of 51.3 nA. Leveraging the low-loss characteristics of ${\mathrm{Si}}_{3}N_{4}$ platforms, researchers at the University of Virginia have advanced the heterogeneous integration of III–V detectors via adhesive die-to-wafer bonding. While Tabatabaei et al. initially validated this approach by integrating MUTC photodiodes onto ${\mathrm{Si}}_{3}N_{4}$ waveguides to achieve high-bandwidth photodetection, achieving a 3 dB bandwidth of 81 GHz [91]. Building on this architecture, the group demonstrated high-coherence millimeter-wave generation at 98 GHz by heterogeneously coupling high-speed photodiodes with on-chip microcavity solitons [92], as shown in Figure 9b. This scalable approach represents a significant step toward realizing large arrays of ultra-stable mmWave sources for next-generation communication and sensing applications.

Beyond polymer-based adhesives, the use of thin dielectric interlayers, particularly ${\mathrm{Al}}_{2}O_{3}$, has been investigated to facilitate robust monolithic integration. Cheng et al. utilized an ${\mathrm{Al}}_{2}O_{3}$ bonding interface to realize InGaAs MSM photodetectors integrated with InP grating couplers on silicon, demonstrating efficient optical coupling and low dark current characteristics [93]. Extending this approach to three-dimensional architectures, Geum et al. achieved the monolithic 3D (M3D) integration of InGaAs photodetectors directly atop SOI-MOSFETs via ${\mathrm{Al}}_{2}O_{3}$ bonding, thereby validating the technique’s potential for high-density, multi-functional imaging systems [94].

Recently, Ke et al. advanced the heterogeneous integration of InGaAs on silicon by introducing a microcrystalline Germanium-mediated bonding technique to mitigate interface-state-induced leakage [73]. This approach yielded high-performance InGaAs PIN photodetectors with a 3 dB bandwidth of 24 GHz and a responsivity of 0.75 A/W at 1550 nm. Furthermore, the authors demonstrated InGaAs/Si APDs exhibiting unprecedented thermal robustness up to 470 K and peak responsivities reaching 42 A/W [95]. Utilizing a two-step bonding variant, the group subsequently achieved a record gain of 235 and an exceptionally low breakdown voltage temperature coefficient of 0.088%, highlighting the potential of this platform for demanding optoelectronic applications [96].

While the aforementioned intermediate layers primarily optimize optical coupling or interface quality, the integration of photodetectors with silicon readout integrated circuits (ROICs) necessitates robust electrical connectivity. Consequently, copper–copper (Cu-Cu) thermocompression bonding has emerged as the dominant enabler for 3D packaging, providing high-density vertical interconnects. Leading this trajectory, Sony utilized Cu-Cu hybridization to vertically stack InGaAs photodiodes onto Si ROICs, realizing back-illuminated image sensors with a fine pixel pitch of 5 µm [97]. Furthermore, imec has significantly advanced the scaling of this 3D integration technology by developing a Cu/SiCN hybrid bonding process that achieves interconnect pitches as small as 1 µm, establishing a foundation for ultra-dense chip-to-wafer stacking [98].

### 3.2. Micro-Transfer Printing

μTP has garnered extensive attention as a transformative heterogeneous integration methodology. This technique harnesses the viscoelastic properties of a polydimethylsiloxane (PDMS) stamp to manipulate pre-fabricated, dense device arrays [99,100]. By leveraging a kinetically controlled adhesion mechanism, μTP facilitates the selective “pick-up” of functional micro-components from a source wafer and their subsequent high-precision printing onto target substrates, such as SOI, SiN, or thin-film lithium niobate (TFLN) platforms [101,102,103,104].

The preeminent merit of μTP lies in the strategic decoupling of device fabrication from system integration. By executing high-density arrayed processing on the source wafer followed by discrete placement onto the target substrate, the technique maximizes material utilization efficiency and curtails production costs [99,105]. Furthermore, the use of multi-tip arrayed stamps facilitates massive parallelization, enabling the simultaneous transfer of thousands of micro-components. Most notably, since devices are fully processed on their native substrate, μTP allows for rigorous pre-screening and wafer-level testing prior to integration.

**Figure 10:**
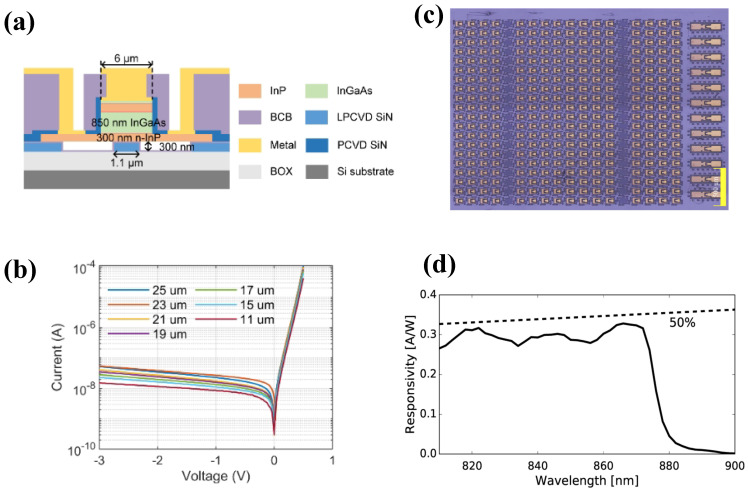
(**a**) Structure of the waveguide-coupled photodiode. (**b**) Dark current. Reproduced with permission from Ref. [77]. (**c**) Microscope images of source substrate with dense PD configuration. (**d**) The obtained waveguide-referred responsivities. Reproduced with permission from Ref. [78].

The InP material system encompasses the prime spectral windows for optical communications, representing the most technologically mature application area for μTP-integrated photodetectors. Qin et al. demonstrated an InGaAs/InP heterojunction MUTC photodetector integrated on Si via μTP [77]. The device exhibited a 3-dB bandwidth of 32GHz and a responsivity of 0.3A/W at 1310nm. Maes et al. reported μTP-integrated UTC-PDs on ${\mathrm{Si}}_{3}N_{4}$ with a 155GHz bandwidth and 0.3A/W responsivity, as shown in Figure 10. The architecture exhibited a dark current below 10nA at −1V, illustrating a viable route for combining III–V active components with low-loss ${\mathrm{Si}}_{3}N_{4}$ platforms for ultra-wideband signal detection.

GaAs primarily covers the 850 nm band, serving as a cornerstone material for short-reach data center interconnects. Goyvaerts et al. demonstrated a GaAs PIN photodetector integrated onto a ${\mathrm{Si}}_{3}N_{4}$ waveguide via μTP [78]. By placing a vertical GaAs detector atop a grating coupler, the device achieved a waveguide-referenced responsivity of 0.3A/W at 860nm, as shown in Figure 10d, corresponding to an external quantum efficiency (EQE) of 47%. The photodetector exhibited excellent electrical characteristics, with a dark current as low as 11.5pA.

Guo et al. demonstrated a μTP-integrated GaInAsSb-GaSb detector [79] on Si with a 1.25A/W responsivity at 2.45μm. The device achieved a 62% EQE, underscoring the potential of μTP for expanding the functional wavelength range of integrated silicon photonic systems into the mid-infrared spectrum.

**Figure 11:**
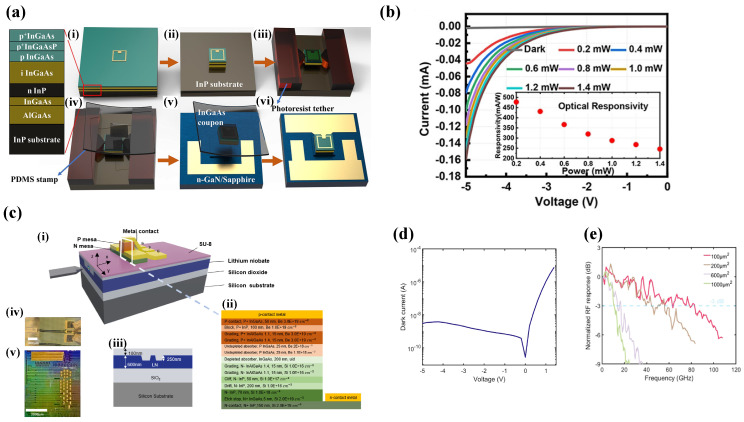
(**a**) Fabrication process of the PD. (i) Evaporate the Ti/Pt/Au electrode of p-InGaAs; (ii) ICP etch to form InGaAs mesa; (iii) lithography to form tether and undercut the InGaAs/AlGaAs release layer; (iv) use PDMS stamp to pick up the InGaAs coupon; (v) transfer print the InGaAs coupon to the middle of n-GaN mesa; (vi) the detector was fabricated after standard fabrication process. (**b**) Photoresponse characteristics. Reproduced with permission from Ref. [81]. (**c**) MUTC PDs integrated on TFLN. (i) Structural illustration; (ii) epitaxial structure of the n-down PD; (iii) TFLN waveguide cross section; (iv) microscope image of a device; (v) microscope image of chip with integrated PDs. (**d**) Dark current. (**e**) Simulations and measurements of bandwidth. Reproduced with permission from Ref. [80].

Being minimally constrained by lattice mismatch, μTP enables heterogeneous integration on substrates that are otherwise incompatible with conventional growth methods. TFLN is a leading platform for high-performance modulators; however, it lacks intrinsic optical sensing capabilities. Guo et al. demonstrated the heterogeneous integration of MUTC photodetectors onto LNOI waveguides via μTP [80]. At 1550nm, the device exhibited an internal responsivity of 0.6A/W and a dark current of 3nA, as shown in Figure 11b. The architecture achieved a 3-dB bandwidth of 80GHz, with high-speed characterization validating 40Gbit/s data reception without external amplification.

Liu et al. reported the heterogeneous integration of InGaAs/InP epitaxial films onto n-GaN substrates via μTP [81]. Through optimized surface treatment and thermal annealing at 420 °C, a high-quality quasi-ohmic contact was established at the InP/GaN interface. Figure 11 shows the resulting photodetector achieved a responsivity of 0.5A/W at 1550nm, demonstrating the capability of μTP to bridge highly dissimilar material systems.

### 3.3. Hetero-Epitaxial

Although bonding provides excellent results, it is constrained by high material costs and process complexity. Additionally, the bonding interface exhibits heightened sensitivity to particulate contamination, necessitating stringent cleanroom protocols. Monolithic integration via direct hetero-epitaxy theoretically offers the ultimate path toward minimal manufacturing costs and maximum integration density [106]; however, this paradigm must reconcile the formidable impediments posed by the lattice mismatch between dissimilar material systems. Two primary methodologies dominate the monolithic integration of III–V on Si. Blanket hetero-epitaxy involves the unrestricted deposition of III–V thin films across the entire Si wafer. Conversely, selective hetero-epitaxy enables the growth of III–V materials within localized regions with high aspect ratios, resulting in III–V crystals with diverse geometries [107,108,109,110]. This latter approach enables site-controlled synthesis and leverages geometric defect trapping to enhance material quality.

#### 3.3.1. Blanket Hetero-Epitaxy

**Figure 12:**
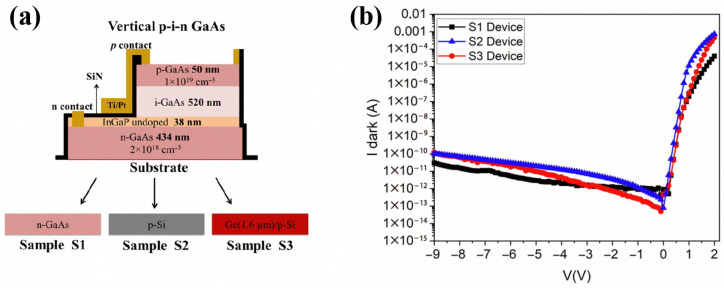
(**a**) Structure of the GaAs-based PD. (**b**) Dark current. Reproduced with permission from Ref. [74].

In blanket heteroepitaxy, the fundamental strategy entails the deployment of buffer layers to suppress the propagation of threading dislocations induced by lattice mismatch, thereby ensuring the synthesis of high-quality, device-grade crystals at the surface. Mehdi et al. demonstrated the successful growth of GaAs p-i-n photodetectors on 300mm Si wafers using blanket hetero-epitaxy [74]. By incorporating a 1.6μm Ge buffer layer, they effectively suppressed the threading dislocation density (TDD) to $3.3\times 10^{7}\;$${\mathrm{cm}}^{-2}$, marking a two-order-of-magnitude reduction compared to direct growth on Si. As shown in Figure 12b, the fabricated device exhibited exceptionally low dark current, remaining below 100 pA under a bias of −9 V at room temperature.

InP-based material systems are indispensable for long-haul optical communications, serving as lattice-matched foundations for InGaAs and InAlAs. While InGaAs provides exceptional responsivity across the O- and C-bands, InAlAs is extensively employed as the multiplication layer in high-performance APDs. Leveraging these material advantages, Sun et al. demonstrated a waveguide-integrated modified uni-traveling carrier (MUTC) photodetector successfully synthesized on Si through direct hetero-epitaxy [75]. By employing a multi-stage buffer architecture comprising Ge, GaAs, and InAlAs, the device achieved a suppressed dark current of 0.1μA at a −3V bias, alongside an internal responsivity of 0.78A/W.

GaSb-based materials are the cornerstone for mid- and far-infrared (MWIR/LWIR) detection; however, the 12% lattice mismatch between GaSb and Si severely hampers monolithic integration. To circumvent this bottleneck, Carrington et al. implemented a sophisticated strain-relief strategy [76]. This involved the formation of an AlSb interfacial misfit (IMF) array, a bimodal temperature growth regimen, and the deployment of dislocation filter superlattices (DFLs) to ensure a high-quality GaSb virtual substrate. Utilizing the optimized buffer scheme, the TDD was limited to $3\times 10^{7}\;{\mathrm{cm}}^{-2}$, enabling broad-spectrum detection from 2 to 6μm. At 200K, the photodetector achieved a responsivity of 0.88A/W and a detectivity of $1.5\times 10^{10}\;\mathrm{Jones}$, representing a significant advancement in monolithic GaSb-on-Si integration.

#### 3.3.2. Selective Hetero-Epitaxy

While blanket hetero-epitaxy has achieved significant milestones, the reliance on multi-micrometer buffer stacks remains a primary constraint. These layers increase epitaxial overhead and, more critically, increase the physical separation between the active devices and the Si waveguides. This separation significantly impairs evanescent field overlap, thereby precluding efficient optical coupling. Selective hetero-epitaxy not only facilitates superior defect filtering and strain relaxation through localized geometric constraints but also enables site-specific synthesis for multi-functional integration [108,111,112]. These inherent advantages have paved the way for several distinct implementation routes.

Aspect Ratio Trapping (ART) serves as a pivotal methodology in selective hetero-epitaxy. Xue et al. demonstrated the monolithic convergence of buffer-less InP/InGaAs nanoridge photodetectors on (001) SOI platforms [107]. By leveraging ART, they achieved high-quality active regions in close proximity to the Si handle. Optoelectronic characterization revealed a peak responsivity of 1.06A/W at 1.55μm, with a broadband spectral response spanning from 1450nm to 1650nm.

The Interuniversity Microelectronics Centre (imec) pioneered nanoridge engineering (NRE) to facilitate seamless material convergence. Ozdemir et al. successfully mitigated epitaxial defects through geometric trapping and InGaP passivation [108], achieving record-low dark currents and a responsivity of 0.65A/W. This work underscores the feasibility of large-scale III–V integration using established silicon manufacturing infrastructure.

Beyond vertical integration schemes like ART and NRE, researchers have pioneered lateral integration methodologies. In this co-planar configuration, the photodetectors are synthesized within the same plane as the Si device layer. This architecture facilitates butt-coupling—where the optical mode is directly injected into the III–V absorption region—enabling superior coupling efficiency and a significantly reduced device footprint compared to vertical evanescent-coupling counterparts.

**Figure 13:**
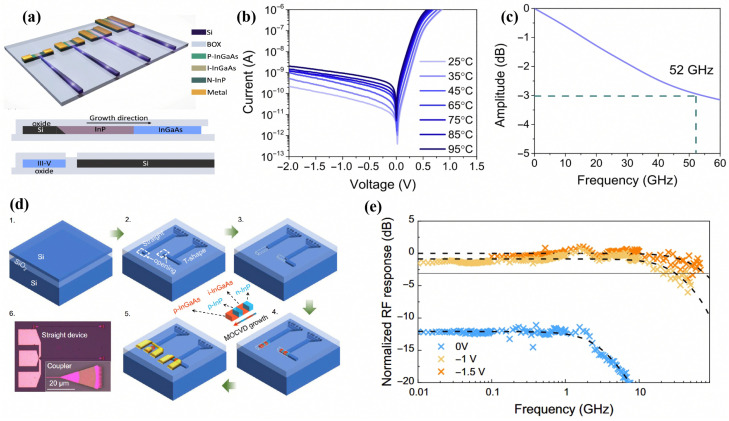
(**a**) Schematic of the Si-waveguide-coupled III–V PD on SOI with various dimensions. (**b**) Dark current of the PD. (**c**) Frequency response. Reproduced with permission from Ref. [109]. (**d**) Simplified schematic of the fabrication process: 1. SOI wafer. 2. Patterned top Si layer and openings. 3. Partial etch-back of Si to form hollow $SiO_{2}$ template with a Si seed. 4. MOCVD of heterostructure p-i-n device, the arrow in the inset shows the growth direction. 5. Ni/Au contacts on the nanostructure. 6. Optical microscope image of a straight device and the coupler in the inset. (**e**) Frequency response. Reproduced with permission from Ref. [110].

Pioneered by Kei May Lau’s group at HKUST, the Lateral Aspect Ratio Trapping (LART) technique represents a breakthrough in co-planar integration. LART facilitates the horizontal proliferation of III–V epilayers across the buried oxide layer of an SOI substrate, achieving a remarkable butt-coupling efficiency of 70%. As shown in Figure 13b, the resulting photodetectors exhibit an ultralow dark current of 60pA and support broadband operation across the entire telecommunication spectrum. Furthermore, the devices demonstrate exceptional high-speed performance, with a 3-dB bandwidth exceeding 52GHz, as shown in Figure 13c.

Another prominent lateral integration methodology is Template-Assisted Selective Epitaxy (TASE). The TASE process involves defining a hollow oxide micro-cavity atop a Si waveguide, with a monocrystalline Si seed preserved at the channel’s extremity. Subsequently, III–V materials nucleate from this seed to conformally fill the oxide cavity. Wen et al. leveraged TASE to achieve the monolithic convergence of co-planar InP/InGaAs heterojunction photodetectors on SOI [110]. Figure 13c shows a 3-dB bandwidth surpassing 70GHz at 1320nm, while high-speed testing validated 100Gbps signal reception alongside a dark current density as low as 0.048 ${A/\mathrm{cm}}^{2}$.

## 4. Two-Dimensional Materials

While III–V integration is well-established, interfacial lattice mismatch remains a persistent challenge. Consequently, atomically thin 2D materials have emerged as a compelling alternative. Unlike traditional semiconductors, 2D materials are characterized by weak van der Waals interlayer forces, which effectively decouple their crystalline structure from the lattice symmetry of the underlying substrate [113,114]. This unique property enables the strain-free integration of various 2D crystals onto silicon photonic platforms, bypassing the conventional limitations of interfacial dislocations. However, 2D materials are highly sensitive to environmental factors due to their atomically thin structures. For example, black phosphorus undergoes rapid oxidative degradation in air. Consequently, their long-term stability and process reliability depend heavily on advanced encapsulation techniques. This section systematically evaluates the integration strategies and performance of emerging 2D candidates, including graphene [115,116], black phosphorus (BP) [117,118,119], and transition metal dichalcogenides (TMDCs) [120,121]. Table 2 summarizes the key performance parameters of various 2D material-based photodetectors.

### 4.1. Graphene

As a zero-bandgap semimetal, graphene enables electronic transitions with near-zero energy requirements, facilitating an ultrabroadband spectral response from ultraviolet to terahertz frequencies [115,116]. Its exceptional carrier mobility, with theoretical limits exceeding 200,000$\;{\mathrm{cm}}^{2}/V\cdot s$, makes it ideal for high-speed optoelectronics. However, the intrinsic optical absorption of monolayer graphene is limited to approximately 2.3%, a primary bottleneck that constrains the responsivity of graphene-based photodetectors. In recent years, the research focus regarding graphene has centered on surmounting the absorption bottleneck without compromising its ultrafast response.

**Figure 14:**
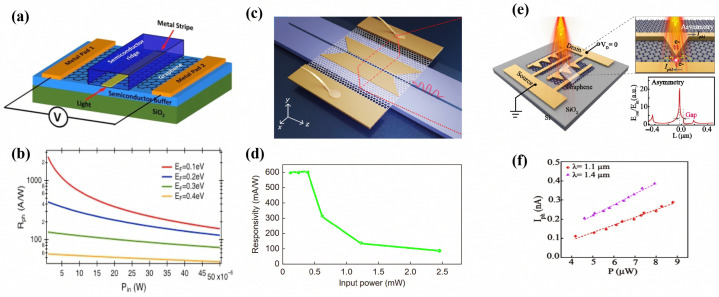
(**a**) Schematic of the plasmonic PB photodetector. (**b**) Responsivity as a function of input power. Reproduced with permission from Ref. [122]. (**c**) Structure of the photodetector based on the double slot structure. (**d**) The responsivity as a function of the input optical power. Reproduced with permission from Ref. [123]. (**e**) Structure of the asymmetric metallic nanostructure-based GPDs. (**f**) Photocurrents as a function of light power. Reproduced with permission from Ref. [124].

Gosciniak and Khurgin proposed a graphene photodetector based on a long-range dielectric-loaded surface plasmon polariton (LR-DLSPP) waveguide [122]. This architecture confines the optical field at the edges of the metal strip, as shown in Figure 14a, generating a strong in-plane electric field component that enhances graphene absorption. Numerical simulations indicated a responsivity of 1100A/W within the 1550nm telecommunication band. Yan et al. reported a novel Si-based graphene photodetector incorporating a double-slot architecture [123]. By integrating a Si slot waveguide with a gold plasmonic slot waveguide, this configuration effectively balances the trade-off between enhanced graphene absorption and metallic ohmic losses. The device attains superior performance within the 1550 nm telecommunication band, yielding a measured responsivity of 0.6A/W, as shown in Figure 14d. Furthermore, the detector achieved a 3 dB bandwidth of 78 GHz and successfully demonstrated the efficient reception of 115 Gbit/s NRZ signals.

Although plasmonic enhancement increases absorption, symmetric architectures typically lead to equal carrier diffusion probabilities under zero bias, resulting in zero net photocurrent. To enable self-powered detection, Zhang et al. engineered an asymmetric nanogap using stripe and triangle arrays on a graphene surface [124]. This design facilitates wavelength selectivity and polarization sensitivity, achieving a responsivity of 25mA/W at 1400nm without external biasing, as shown in Figure 14f.

### 4.2. Black Phosphorus

Black phosphorus (BP) is a layered semiconductor characterized by an intrinsic direct bandgap across all thicknesses [117]. Its energy gap undergoes a layer-dependent reduction, narrowing from 1.7eV in monolayers to 0.3eV in the bulk form [119,125,126]. This property, combined with its high carrier mobility, facilitates efficient light-matter interaction and high-speed photo-response.

**Figure 15:**
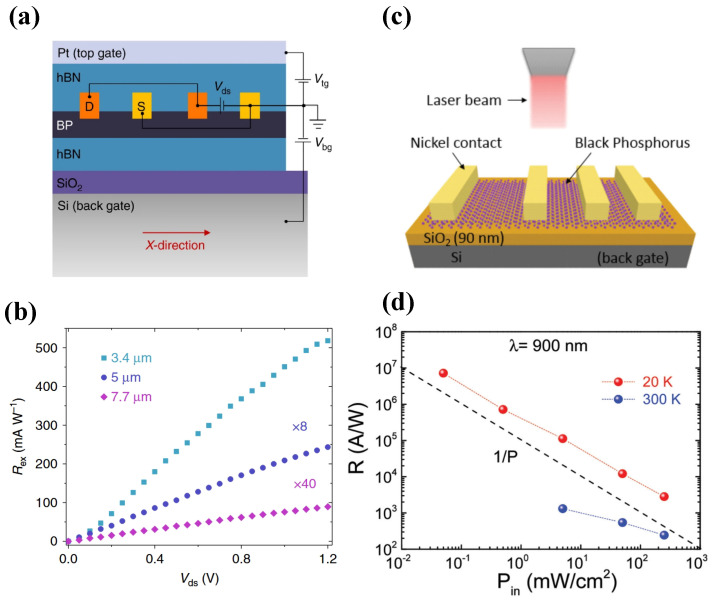
(**a**) Structure of the tunable BP mid-IR photodetector based on a dualgate transistor configuration. (**b**) Photo-responsivity as a function of source-drain bias. Reproduced with permission from Ref. [127]. (**c**) Schematic view of the device. (**d**) The responsivity as a function of the input optical power. Reproduced with permission from Ref. [118].

Chen et al. demonstrated a dual-gated BP heterojunction photodetector encapsulated in hexagonal boron nitride (hBN) [127]. The application of a vertical displacement field enabled a significant extension of the detection cut-off wavelength for 10-layer BP flakes, shifting the threshold from 3.7μm to over 7.7μm, as shown in Figure 15b. This bandgap-tuning strategy allows BP to operate in the LWIR regime, surpassing its intrinsic bulk limit. Huang et al. reported a back-gated field-effect transistor photodetectors using approximately 15 layers of mechanically exfoliated BP [118]. Figure 15d shows that the device exhibited a broad spectral response from 400nm to 900nm, achieving a responsivity of $4.3\times 10^{6}\;A/W$ at 300K. This work establishes a significant performance benchmark for BP-based detectors in ultra-sensitive, broadband optoelectronic applications.

Due to its puckered honeycomb lattice, BP exhibits significant optical anisotropy, making it an ideal candidate for polarization-sensitive detection. To amplify this effect, Wang et al. integrated low-contrast gratings with a BP-on-SOI photodetector [128]. This strategy achieved a responsivity of 9.13mA/W at 1514nm, while simultaneously improving the polarization extinction ratio from 2.3 to 13.1.

### 4.3. Transition Metal Dichalcogenides

Transition metal dichalcogenides (TMDCs) are characterized by the chemical formula ${\mathrm{MX}}_{2}$, where M represents a transition metal (e.g., Mo, W) and X denotes a chalcogen (e.g., S, Se, Te) [120,121]. The electronic properties of TMDCs are highly dependent on their specific atomic composition and layer count [129]. This versatility enables the TMDC family to span a broad spectral range, encompassing wide-bandgap semiconductors such as ${\mathrm{WS}}_{2}$[130], narrow-bandgap variants like ${\mathrm{MoTe}}_{2}$[131,132] and ${\mathrm{MoS}}_{2}$[133], and even semimetals such as ${\mathrm{PtSe}}_{2}$ [134].

${\mathrm{MoS}}_{2}$ are promising for on-chip optoelectronics due to their thickness-dependent bandgaps and strong light-matter interaction. While bare ${\mathrm{MoS}}_{2}$ photodetectors offer high gain via photoconductive mechanisms, they suffer from large dark currents and slow speeds caused by persistent photoconductivity (PPC) and surface traps. By utilizing ${\mathrm{MoS}}_{2}$-based heterostructures, the engineered built-in electric field at the van der Waals interface enables rapid charge separation and microsecond-scale response [133,135]. Yang et al. demonstrated a waveguide-integrated ${\mathrm{MoTe}}_{2}/{\mathrm{MoS}}_{2}$ vdW heterojunction photodetector on a TFLN platform [132]. By combining the NIR sensitivity of ${\mathrm{MoTe}}_{2}$ with the visible-light response of ${\mathrm{MoS}}_{2}$, the device achieved an ultralow dark current of less than 150pA at −1V and a high optical on/off ratio, alongside robust detection at 1310nm.

**Figure 16:**
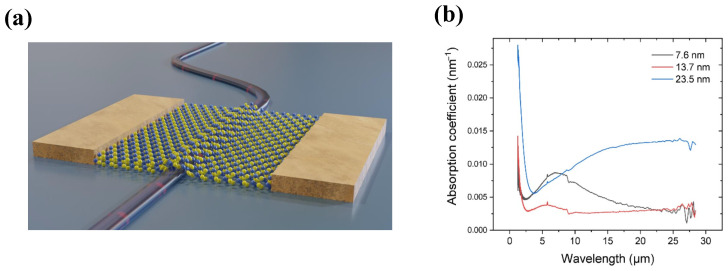
(**a**) Schematic view of the device. (**b**) Absorption coefficients of ${\mathrm{PtSe}}_{2}$ films with different thicknesses. Reproduced with permission from Ref. [136].

${\mathrm{PtSe}}_{2}$ is distinguished by its layer-dependent bandgap and exceptional environmental stability, exhibiting semimetallic behavior in thicker films. Xu et al. demonstrated optoelectronic integration using ten-layer ${\mathrm{PtSe}}_{2}$ on a 70 nm ultrathin silicon racetrack microring resonator, achieving an enhanced absorption coefficient of 0.577dB/μm at 2200nm[134]. Furthermore, in 2022 Parhizkar et al. validated the conformal growth of ${\mathrm{PtSe}}_{2}$ directly on silicon waveguides [136], yielding a responsivity of 11mA/W at 1550nm with a fast response time of 8.4μs. Notably, ${\mathrm{PtSe}}_{2}$ maintains significant absorption into the LWIR regime, with a spectral range extending to 28μm, as shown in Figure 16b.

The inherent flexibility and tunable electronic properties of 2D materials have paved the way for a new generation of reconfigurable neuromorphic vision systems. Unlike traditional architectures that rely on rigid hardware and complex algorithms for functional switching, 2D material-based devices demonstrate superior reconfigurable characteristics through the direct modulation of physical processes at the atomic scale. Specifically, by leveraging oxygen-mediated surface states in ${\mathrm{PtSe}}_{2}$ [137] or the synergistic coupling of positive and negative photoconductivity in ${\mathrm{MoS}}_{2}/{\mathrm{PtSe}}_{2}$ heterostructures [138], these devices can autonomously reconfigure their responsivity to mimic biological functions such as spontaneous chromatic adaptation and locust-inspired collision detection. Furthermore, the integration of 2D van der Waals heterojunctions like b-AsP/${\mathrm{MoTe}}_{2}$ enables an all-optically triggered reconfigurability in the MIR regime [139]. By dynamically adjusting the statistical parameters of external optical sampling signals, the systems can flexibly reconfigure their dynamic range and encoding precision to suit diverse application scenarios, ranging from high-sensitivity medical imaging to wide-range industrial monitoring.

While demonstrating superior optoelectronic figures of merit, the practical deployment of these emerging material-based photodetectors remains contingent upon addressing critical engineering bottlenecks, including their CMOS process compatibility, large-scale fabrication uniformity, and the environmental robustness of 2D layers against long-term ambient degradation.

**Table 2:** Performance summary of 2D materials photodetectors.

Device Type	Wavelength Range (μm)	Responsivity (A/W)	Rise, Fall Time	Ref.
${\mathrm{MoTe}}_{2}$	0.413–1.1	88	330 μs	[140]
${\mathrm{PdTe}}_{2}$	1000–7500	10	1 μs, 2.2 μs	[141]
${\mathrm{TaIrTe}}_{4}$	0.532–10.6	$2.0\times 10^{-5}$	27 μs	[142]
${\mathrm{PtTe}}_{2}$	0.200–1.65	0.406	7.51 μs, 36.7 μs	[143]
${\mathrm{MoS}}_{2}$	0.5–1.1	1.358	710 μs, 660 μs	[144]
${\mathrm{Bi}}_{2}{\mathrm{Te}}_{3}$	0.532–1.55	35	8.7 ms	[145]
${\mathrm{WSe}}_{2}/{\mathrm{SnS}}_{2}$	0.4–0.9	244	13 ms, 24 ms	[146]
${\mathrm{WS}}_{2}$	0.457–0.647	$9.2\times 10^{-5}$	5.3 ms, 5.3 ms	[147]
${\mathrm{MoS}}_{2}/{\mathrm{MoTe}}_{2}$	0.560–1.55	0.046	60 μs, 25 μs	[148]
BP	0.532–3.39	82	130 μs	[149]
BP	2.5–3.7	0.047	-	[150]
BP	3.7–7.7	0.518	-	[127]
BP	0.31–0.95	$9\times 10^{4}$	1 ms, 4 ms	[151]
BP	0.4–3.75	$1.5\times 10^{-3}$	<40 μs	[152]
Graphene	0.375–118	$1.5\times 10^{-3}$	∼100 ms	[153]
Graphene	1.55	$∼0.5\times 10^{-3}$	500 GHz	[154]
Graphene	1000	$R_{v}∼0.15$ V/W	-	[155]
Graphene	0.457–0.785	0.01	-	[156]
Graphene	8–220	$∼5\times 10^{-9}$	50 ps	[157]
Graphene/AlSb/InSb	0.2–1.4	0.329	13 ns,11 ns	[158]
${\mathrm{PtSe}}_{2}/{\mathrm{MoS}}_{2}$	0.405–0.980	4.52	20 ms	[159]
${\mathrm{PdSe}}_{2}$ p-i-n	1.064	1.1	3 μs, 6 μs	[160]
Graphene/${\mathrm{PtSe}}_{2}$/${\mathrm{SiO}}_{2}$/Si	0.375–1.55	0.572	17.3 μs, 38.8 μs	[161]

## 5. Discussion

The rapid evolution of silicon-based on-chip infrared photodetectors reflects a fundamental paradigm shift from discrete, bulky instrumentation to integrated, SWaP-constrained sensing ecosystems. As reviewed in this work, the field has bifurcated into three distinct yet complementary material platforms: Group IV (Ge/GeSn), III–V compounds, and emerging 2D materials. A critical comparative analysis of these technologies reveals that the choice of platform is increasingly dictated by the specific trade-offs between performance, spectral coverage, and manufacturing scalability. The carrier transport dynamics and band alignment strategies are fundamental to the performance disparities observed among the three material platforms. Group IV materials often face constraints in noise performance and carrier transport, arising from their relatively weak optical absorption, the need for thicker absorption layers, and limited band structure flexibility compared to direct-bandgap III–V compounds. III–V compounds achieve high mobility and quantum efficiency due to their direct bandgaps and small effective masses. However, they are primarily limited by shot noise arising from relatively high dark currents; an exception is GaN, which utilizes its wide bandgap to achieve exceptionally low dark currents and high-sensitivity ultraviolet detection [162,163]. Two-directional materials offer superior electrostatic control to suppress short-channel effects. Their atomic thickness makes them highly sensitive to environmental scattering and contact noise. In graphene-based electronic devices, the noise is predominantly governed by carrier mobility fluctuations, which can be described by the empirical Hooge’s relationship and are sensitive to both intrinsic scattering mechanisms and extrinsic substrate-induced disorders. In contrast, the noise performance of black phosphorus is characterized by a complex interplay between 1/f flicker noise and generation-recombination (G-R) processes, both of which are critically modulated by thickness-dependent band structures and surface traps resulting from ambient-induced degradation. For TMDC-based devices, the noise characteristics are primarily governed by the high contact resistance at the metal-semiconductor interface and the carrier number fluctuation mechanism. To provide a clearer comparative perspective, Table 3 contrasts the performance of the discussed material platforms across several key metrics, including responsivity, detectivity, and operating temperature.

Ge-on-Si photodetectors have matured into the industrial applications for the telecommunication bands, achieving a high degree of CMOS compatibility. The architectural innovations discussed, such as inductive gain peaking and lateral PIN configurations, have effectively pushed the bandwidth-responsivity envelope. However, the intrinsic bandgap of Ge limits its efficacy beyond 1.6 μm. While GeSn alloys offer a monolithic solution to extend detection into the MWIR, achieving high-quality epitaxial growth with high Sn concentrations remains a challenge due to compressive strain and segregation issues. In contrast, III–V materials remain the benchmark for high-performance sensing, offering superior quantum efficiency and lower dark currents compared to Group IV alternatives. Through bonding, hetero-epitaxy, and μTP, III–V compound PD integrated with Si platforms offer distinct benefits in optical response and wavelength coverage. While heterogeneous bonding serves as a robust solution for lattice-mismatched integration in silicon photonics and 3D packaging, the stringent requirement for specialized equipmen limits its high-volume manufacturing. Alternatively, hetero-epitaxy promises seamless monolithic integration but requires overcoming substantial hurdles related to buffer layer thickness, material defects, and optical coupling performance. μTP delivers high flexibility and low costs, proving particularly advantageous for complex silicon photonic systems. This emerging technique serves as a versatile middle ground, combining the material quality of native substrates with the integration flexibility of heterogeneous processing. Its ability to integrate diverse materials allows for an excellent approach to system design. However, μTP remains constrained by high process complexity and lack of a eco-system. While 2D materials offer a disruptive alternative-bypassing lattice mismatch constraints through van der Waals integration and enabling unique functionalities like polarization sensitivity and zero-bias operation, they currently suffer from immature large-scale fabrication processes and contact resistance issues that impede their transition from laboratory to foundry.

The trajectory of integration methodologies highlights a clear trend toward decoupling material quality from substrate constraints. Looking forward, the development of Si-based infrared photodetectors must address several system-level challenges to enable extensive deployment. First, realizing room-temperature operation with suppressed dark current is critical to eliminating the reliance on bulky cooling systems. Simultaneously, as component density increases, future architectures will likely leverage 3D stacking to minimize parasitic capacitance and enable massive parallel readout, essential for high-frame-rate imaging and high-capacity data links. Ultimately, the cross-disciplinary convergence of these hardware breakthroughs with artificial intelligence and IC design will propel the field beyond simple optoelectronic conversion toward comprehensive system integration, enabling the realization of next-generation, low-cost, and reliable smart optoelectronic chips.

## Figures and Tables

**Table 3:** Comparative analysis of Silicon-based infrared photodetectors across key material platforms.

Key Metrics	Group IV (Ge/GeSn)	III–V Compounds	2D Materials
Responsivity	High	Very High	Variable
Detectivity	Moderate	High	Lab High; Noise limited
Operating Temp.	RT (Ge); Cooling (GeSn)	RT (SWIR); Cooling (MWIR)	Specific RT; High dark current
CMOS Compatibility	Excellent (Monolithic)	Moderate (Heterogeneous)	High (vdW); Lacks standards
Spectral Range	O/C bands to SWIR	SWIR to LWIR	Ultra-broadband

## Data Availability

Data is contained within the article..
